# The Vastus Lateralis Muscle Interstitium Proteome Changes after an Acute Nociception in Patients with Fibromyalgia Compared to Healthy Subjects—A Microdialysis Study

**DOI:** 10.3390/biomedicines11010206

**Published:** 2023-01-13

**Authors:** Bijar Ghafouri, Daria Matikhan, Nikolaos Christidis, Malin Ernberg, Eva Kosek, Kaisa Mannerkorpi, Björn Gerdle, Karin Wåhlén

**Affiliations:** 1 Pain and Rehabilitation Centre, Department of Health, Medicine and Caring Sciences, Linköping University, SE-581 85 Linköping, Sweden; 2 Department of Dental Medicine, Karolinska Institutet, the Scandinavian Center for Orofacial Neurosciences (SCON), SE-141 04 Huddinge, Sweden; 3 Department of Clinical Neuroscience, Karolinska Institutet, SE-171 77 Stockholm, Sweden; 4 Department of Surgical Sciences, Uppsala University, SE-751 85 Uppsala, Sweden; 5 Department of Neuroscience and Physiology, Section of Health and Rehabilitation, Physiotherapy, Sahlgrenska Academy, University of Gothenburg, SE-405 30 Gothenburg, Sweden; 6 Centre for Person Centered Care (GPCC), Sahlgrenska Academy, University of Gothenburg, SE-405 30 Gothenburg, Sweden

**Keywords:** biomarkers, chronic pain, interstitial fluid, muscle proteome, painful muscle

## Abstract

Fibromyalgia (FM) is a complex disorder and a clinical challenge to diagnose and treat. Microdialysis is a valuable tool that has been used to investigate the interstitial proteins and metabolites of muscle in patients with fibromyalgia. The implantation of the catheter in the muscle causes acute tissue trauma and nociception. The aim of this study was to investigate acute proteome changes in the vastus lateralis muscle in women fibromyalgia patients (FM) and healthy subjects (CON). A further aim was to study if a 15-week resistance exercise program in FM had any influence on how chronic painful muscle responds to acute nociception. Twenty-six women patients with FM and twenty-eight CON were included in this study. A microdialysis catheter (100 kilo Dalton cut off, membrane 30 mm) was inserted in the vastus lateralis muscle, and samples were collected every 20 min. Subjects rated pain before catheter insertion, directly after, and every 20 min of sample collection. Dialysate samples from time points 0–120 were pooled and considered trauma samples due to the catheter insertion. The samples were analyzed with nano-liquid chromatography-tandem mass spectrometry (nLC-MS/MS). Advanced multivariate data analysis was used to investigate protein profile changes between the groups. Multivariate data analysis showed significant (CV-ANOVA *p* = 0.036) discrimination between FM and CON based on changes in 26 proteins. After the 15-week exercise intervention, the expression levels of the 15 proteins involved in muscle contraction, response to stimulus, stress, and immune system were increased to the same expression levels as in CON. In conclusion, this study shows that microdialysis, in combination with proteomics, can provide new insights into the interstitial proteome in the muscle of FM. In response to acute nociception, exercise may alter the innate reactivity in FM. Exercise may also modulate peripheral muscle proteins related to muscle contraction, stress, and immune response in patients with FM.

## 1. Introduction

Fibromyalgia (FM) is characterized by symptoms such as chronic and diffuse musculoskeletal pain. Chronic pain is particularly complicated, involving a complex triad of biological, psychological, and social factors that modulate pain [1]. It is also frequently associated with fatigue, sleep disorders, stiffness of joints and muscles, cognitive impairment, depression, anxiety, and significantly impaired function and quality of life, altogether leading to limitations in daily life [2,3,4,5]. Patients with FM are more likely to have a history of other syndromes, such as irritable bowel syndrome, endometriosis, dysmenorrhea, and headaches, as well as other chronic pain conditions, e.g., rheumatoid arthritis, lupus, and osteoarthritis [6]. The prevalence of FM is estimated to be around 2–4% in the general adult population, depending on classification criteria [7,8], with women being an overrepresented group.

Patients with FM have proven to be less physically active, even though exercise is commonly recommended for pain management [9]. Evidence demonstrates that strength training, as well as aerobic exercise, has positive implications on the clinical picture of FM by affecting general clinical symptoms, such as pain intensity, fatigue, muscle strength, quality of life, and physical functioning, which lead to improvement of daily life [10,11,12,13].

The molecular mechanisms behind FM and the correlation between the molecular network and clinical symptom profile are mainly unknown. Exploring biomarkers that correlate with patients’ symptoms may be valuable for the future improvement of diagnostic tools and treatment for patients with FM. Today there are no clinically suitable objective tests that have been validated for FM. Several studies have investigated the numbers of compounds in serum, plasma, muscle microdialysate, muscle biopsies, and cerebrospinal fluid of patients with FM to find applicable biomarkers of the disease or disease activity. Altered levels of metabolites [14,15,16,17], pro- and anti-inflammatory cytokines [18,19], neuropeptides [20,21], and activation of the *NLRP3* (NOD-, LRR- and pyrin domain-containing protein 3) inflammasome and the consequent release of pro-inflammatory cytokines [22] are examples of what has been studied and identified in patients with FM.

Microdialysis (MD) is a sampling technique that can be used to detect potential biomarkers locally from the interstitial fluid of muscle in humans and animals. This technique involves the exchange of substances via diffusion between the tissue and a semi-permeable catheter membrane that is inserted into the muscle [23]. The catheter insertion causes tissue trauma, which can be studied as a model of acute nociception in the painful human muscle. Increased levels of serotonin have previously been reported in FM in response to catheter insertion [24]. Biochemical changes can be collected before it is diluted in the circulatory system with continuous sampling of compounds in the interstitial space of the muscle.

Proteomics is a useful technique to identify biomarkers of pain conditions, but few studies have investigated the proteome in the muscle interstitium with MD. The interstitial fluid proteome has previously been studied in healthy controls reflecting the protein changes after probe insertion and acute tissue trauma [25]. Furthermore, we have previously explored the trapezius muscle proteome among chronic widespread pain patients showing alterations of proteins associated with muscle damage, muscle recovery, stress, and inflammation [26]. To the best of our knowledge, there are no studies that have investigated the effect of resistance exercise on the interstitial fluid proteome after acute nociception in muscle in FM patients.

To establish MD as a reliable clinical routine technique for in situ monitoring of protein biomarkers and to explore new potential biomarkers for FM, there is a need for a deeper understanding of the muscle proteome and mechanisms that potentially are related to peripheral changes. In our previous studies from the same cohort using targeted analysis to explore circulating and peripheral levels of cytokines [27,28], metabolites [29], and proteins [30,31], we have shown changes/normalizations of biological markers in FM compared with healthy pain-free controls (CON) after 15-weeks of resistance exercise. Therefore, we hypothesized that the protein expression level, after acute nociception, will be different in patients with FM in comparison with CON before an exercise intervention and that these differences can be normalized after a 15-week resistance exercise program. The purpose of this study was to get further insights into the underlying peripheral molecular mechanism of FM. Hence, the aim of this study was to investigate acute proteome changes in the vastus lateralis muscle in FM patients compared to CON. The secondary aim was to investigate the effects of 15-week resistance exercise training on the proteome response to acute nociception in these two groups of subjects.

## 2. Material and Methods

### 2.1. Recruitment Process and Participants

We have previously reported in detail the recruitment process of this randomized multicenter study (Data base: ClinicalTrials.gov identification number: NCT01226784) [26,28,29,32]. In brief, the planning of the study was initiated in 2009, and the recruitment of participants started in 2010, with the completion of data collection in 2013. All participants were women between 20 and 65 years. Additional criteria for FM patients were a clinical assessment confirming the diagnosis of FM according to the American College of Rheumatology (ACR) 1990 [33]. For the CON group, no present pain was required. The exclusions criteria for both groups were a blood pressure >160/90 mmHg, a primary cause of pain other than FM (e.g., osteoarthritis or rheumatoid arthritis), participation in a rehabilitation program within the past year, regular resistance exercise or relaxation therapy twice a week or more, inability to speak or understand Swedish, high consumption of alcohol, and severe somatic or psychiatric disorders. The participants were not allowed to use analgesics, NSAIDs (Non-steroidal anti-inflammatory drugs), or hypnotics 48 h prior to examination.

Briefly, out of the included participants in the randomized resistance exercise group, 67 FM and 32 CON volunteered to additionally participate in the MD experiment, and out of these participants, 32 FM and 32 CON were allocated to the MD experiment. Due to experimental difficulties and volume restriction of MD dialysate to perform proteomic analysis, 26 FM and 28 CON at baseline and follow-up samples from 25 FM and 26 CON post-exercise were included in this study.

### 2.2. Ethics

The study was approved by the regional ethics committee in Stockholm (Dnr: 2010/1121-31/3). The study was performed in accordance with the Declaration of Helsinki, and after receiving verbal and written information about the study, all participants signed a consent form. The participants were economically compensated for taking part in the intervention and the MD experiment after finalizing the study.

### 2.3. Background Data and Patient-Reported Outcome Measures

In this study, we used subjective measures (patient-reported outcome measures using questionnaires), objective measurements (functional test for assessing muscle strength and capacity), and exploratory proteomic analysis of MD samples from women with FM and CON before and after 15 weeks of resistance exercise.

Anthropometric data such as age (years), weight (kg), height (m), blood pressure (mmHg), and pain duration (years) were collected during the clinical examination (only before the start of the intervention) for all participants. Body mass index (BMI) was calculated as weight/height^2^.

The patient-reported outcome measures have been reported previously [12,28,32,34]. In this study, the following measures were included: the two subscales of the Hospital Anxiety and Depression Scale (HADS) [35], whole body pain intensity using the Visual Analogue Scale (VAS, 0 = no pain, 100 = worst imaginable pain) [36], Pain Disability Index (PDI), Pain Catastrophizing Scale (PCS) [37], Fibromyalgia Impact Questionnaire (FIQ) [38], the psychological and mental summary components of Health Questionnaire 36 (SF-36) [39,40].

Different functional tests were used to assess physical strength and capacity before and after 15 weeks of resistance exercise in both FM patients and CON. These functional tests consisted of a 6-min walk test (6MWT), handgrip force (Newton (N) Grippit, AB Detektor, Gothenburg, Sweden), maximal elbow flexion ((Kg) Isobex Medical Device Solutions AG, Oberburg, Switzerland), and isometric knee extension ((Kg) Steve Strong, Stig Starke HBI, Gothenburg, Sweden)). For more information and discussion about these tests, see previous publications [12,28,29,34]. Furthermore, pressure pain thresholds (PPT) were measured using an algometer (Somedic Sales AB, Höör, Sweden) to estimate pain sensitivity over eight out of 18 tender points according to ACR 1990 criteria. The mean PPT value (kPa) of the eight points was calculated and used in further statistical analysis. The PPT measurement was conducted approximately one week before the microdialysis experiment. For more details about the measurements, see [26].

### 2.4. Resistance Exercise Program

The resistance exercise program has, in detail, been described in our previous studies [12,29,34]. Briefly, the CON and the FM group were assigned a resistance exercise program that lasted for 15 weeks. During these weeks, exercise was performed twice a week under the supervision of experienced physiotherapists. The goal of the resistance exercise program was to improve muscle strength without the risk of causing increased pain when loading the muscles. The intervention comprised an individual introductory meeting in which the participant could describe earlier experiences and thoughts on exercise. During the introductory meeting, exercise instructions, testing, and adjustments of loads were also evaluated. Each session started with a 10 min warm-up followed by 50 min of resistance exercise training using machines or free weights. A larger group of muscles located in the lower extremities, trunk, and arms were preferably targeted during the exercise. At the beginning of the exercise program, the participants were asked to perform one repetition maximum (1RM). On this basis, the participants initiated resistance training at 40% of 1RM, which they would increase to 70–80% of 1RM during the training program. The exercise program was initiated with low loads to promote a sense of control and avoid adverse effects related to exercise [12,29,34]

### 2.5. Microdialysis Procedure

A full description of the MD experiment can be found in our previous publications on the same cohort [27,28,29]. Briefly, MD was performed before the start of the exercise intervention and after 15 weeks of resistance exercise. The participants were instructed not to perform any exercise two days prior to the MD session. They were asked not to drink any beverages that contain caffeine and not smoke on the day the MD was performed. Additionally, they were instructed not to intake analgesics, such as paracetamol or non-steroidal anti-inflammatory drugs, one week before the MD session. All subjects reported that they had followed the instructions. On the day of the study, the participants were allowed to eat breakfast before arriving at the clinic, but they were not allowed to eat during the first 120 min of the MD session. They received a standardized meal at the end of the trauma period (after 120 min).

Before the MD catheter insertion, the skin and subcutaneous tissue were anesthetized with a local injection of 0.5 mL Xylocain (Xylocain^®^ 20 mg/mL, AstraZeneca, Södertälje, Sweden) without adrenaline at the entrance point of the MD catheter. During the local injection, it was important not to anesthetize the underlying muscle. Two types of catheters were used: MD catheters with cut-off points of 100 kDa and 20 kDa (M Dialysis AB, Stockholm, Sweden, membrane 30-mm length, 0.5-mm diameter), where the 100 kDa MD catheter was used for proteomic analysis. Both catheters were inserted into the vastus lateralis muscle parallel to the muscle fibers at a distance between the trochanter and the knee. Ringer Acetate (Fresenius Kabi AB, Uppsala, Sweden) was perfused using a high-precision syringe pump (CMA 107, M Dialysis AB, Stockholm, Sweden) at a rate of 5 µL/min. To mimic the biochemical milieu in the muscle, 3 mM glucose and 0.5 mM lactate were added to the perfusion fluid. Once the catheters were inserted into the muscle, the participants were asked to rest comfortably in a supine position on a bed for 120 min. Samples were obtained every 20 min by changing MD vials, and at the same time, subjects rated their overall pain intensity using the VAS. The MD experiment lasted for approximately 4 h and was divided into four phases: trauma (the first 2 h to stabilize the muscle after catheter insertion), baseline (20 min), work (20 min of dynamic muscle contractions), and recovery (60 min), as described previously [27]. All collected vials were placed on ice during the experiment before they were transferred to Eppendorf tubes and stored at −86 °C until analysis. After 15 weeks of resistance exercise, the MD procedure was repeated in the same muscle. Hence, in this study, we have only analyzed the MD samples from the trauma phase (0–120 min) before and after the intervention.

### 2.6. Protein Extraction and Digestion

Due to the low sample volume and protein concentration in each MD vial, all time points from the trauma phase were pooled (6 vials in total) to be able to run proteomic analysis.

On the day of the analysis, the collected samples were thawed and subjected to a 3 kDa Amicon-spin filter (Merck Millipore, Darmstadt, Germany) to desalt and concentrate the proteins in the samples according to the manufacturer’s recommendation. The desalted proteins were subsequently dried by a speed vacuum concentrator (Savant, Farmingdale, NY, USA), then redissolved in urea buffer solution (8 M urea in 25 mM ammonium bicarbonate) to denature the proteins and incubate at room temperature for at least 30 min. The proteins were then incubated for 15 min in dithiothreitol (25 mM) to reduce disulfide bonds between cysteine residues of proteins and alkylated with iodoacetamide (75 Mm) for an additional 15 min. Before adding trypsin for digestion of protein into shorter peptides, the samples were diluted 8 times with ammonium bicarbonate and filtered with 3 kDa Amicon spin-filter to remove excess salts and urea that interfere with enzyme activity of trypsin and protein concentration was then measured using a 2D quant kit (GE Healthcare, Uppsala, Sweden) according to the manufacturer’s recommendation. A concentration of 0.2 µg/µL of trypsin in a ratio of 1:25 *w*/*w* trypsin/protein was used, and samples were incubated at 37 °C overnight. After tryptic digestion, the samples were dried in a speed vacuum concentrator and stored at −86 °C until analysis. On the day of the analysis, the dried tryptic samples were reconstituted in 0.1% formic acid in MilliQ water, then subjected to mass spectrometry analysis.

### 2.7. Proteomic Analysis

The samples were analyzed using liquid chromatography-tandem mass spectrometry (EASY-nLC-MS/MS, Thermo Scientific, Waltham, USA). The digested proteins containing the fragmented peptides were initially separated by reverse phase high-pressure liquid chromatography (HPLC) on a C18 pre-column (20 mm × 100 μm) followed by a C18 column (100 mm × 75 μm) with particle size 5 μm (NanoSeparations, Nieuwkoop, Netherlands) at a flow rate of 300 nL/min. The gradient buffers contained 0.1% formic acid in water (buffer A) and 0.1% formic acid in acetonitrile (buffer B), and a linear gradient from 0% to 100% of buffer B was used for 90 min to separate the peptides. Then the peptides were ionized with electrospray ionization using an automated online analysis with an LTQ Orbitrap Velos Pro hybrid mass spectrometer (Thermo Scientific) [24]. The acquired raw MS files were searched using the software MaxQuant v. 1.5.8.3 (Max Planck Institute of Biochemistry, Martinsried, Germany) against the Uniprot Human database (downloaded 2019). The following search parameters were used: trypsin as digestion enzyme; the maximum number of missed cleavages 2; fragment ion mass tolerance 0.50 Da; parent ion mass tolerance 6.0 ppm; fixed modification carbamidomethylation of cysteine. Data were filtered at a 1% false discovery rate. The label-free quantitative (LFQ) values were normalized in the software and used for further statistical analysis.

### 2.8. Statistics

Background and questionnaire data were compared between groups using a Mann–Whitney U test and within groups using a Wilcoxon signed rank test (IBM SPSS Statistics, version 28.01.01). A *p*-value < 0.05 was considered significant.

Multivariate data analysis (MVDA) was used to analyze the large set of data that was generated from the proteomic analysis. This was performed using SIMCA-P+ (version 16.0; Sartorius Stedim Biotech, Umeå, Sweden) as previously described [30,41] and according to Wheelock and Wheelock [42]. A principal component analysis (PCA) and an orthogonal partial least square discriminant analysis (OPLS-DA) were performed. PCA is an unsupervised analysis that assesses the dataset’s quality and homogeneity and can highlight eventual outliers in the dataset.

Furthermore, OPLS-DA was performed to investigate the multivariate correlations between the proteins and the observations (FM vs. CON). The data were mean-centered and scaled for unified variance (UV-scaled). The OPLS-DA was performed in two steps. First, a model was built including all the proteins, and from this model, proteins with a variable influence on projection (VIP) value > 1.0 were used in a new model. The VIP value describes the importance and relevance of each X-variable (quantified proteins), pooled over all dimensions, and Y-variables (observations), which included the set of variables that best described Y [26,27]. In tables and plots, the VIP predictive (VIP_pred_) value was used, which described the VIP value for the first predictive component. A higher VIP_pred_ value indicated a more important regressor for the model, where a VIP_pred_ > 1.0 was considered a significant protein.

Cross-validated analysis of variance (CV-ANOVA) *p*-value was calculated for each model as a measure of significance for the model. The multivariate regression was considered significant if the *p*-value was <0.05.

Furthermore, R^2^ and Q^2^ were calculated. The R^2^ value indicated how well the model explains the dataset, while Q^2^ represented a measure of the predictive power of the model. A difference of more than 0.3 indicated that the model’s robustness was poor. The x-variables (proteins) were expressed as loadings in the loading plots and had a value ranging from −1 to +1. The p(corr) is the x-variable loadings scaled as correlation coefficient and range from −1 to +1. The closer p(corr) is to 1, the stronger the correlation [43].

### 2.9. Bioinformatics

An online database tool, STRING version 11.0, was used to create protein-protein networks to analyze the functions of proteins and their interactions **(**https://string-db.org/ (accessed on 22 June 2022). The accession numbers from significant proteins with a VIPpred >1.0 were submitted in the search engine with the following parameters: Organism Homo Sapiens, interaction score was set to high confidence (0.700), the maximum number of interactions was query proteins only, a PPI enrichment *p*-value ≤ 0.01 and a false discovery rate (FDR) of ≤0.05 was set for the Biological Processes. With the confidence mode set, the thickness of the lines in the network indicated the confidence predictions of the interactions [44].

## 3. Results

### 3.1. Background Data

Background data for the patients and healthy controls are summarized in Table 1 and Supplementary Table S1. No significant differences were found between the groups in age, systolic, and diastolic blood pressure. However, the FM group had a significantly higher BMI compared with CON. There were five patients with obesity (BMI > 30), six patients with high BMI (30 > BMI > 25), and ten patients with BMI < 25, while almost all CON had BMI < 25 (*n* = 21).

### 3.2. Patient-Reported Outcome Measures and Muscle Functional Measures

**Figure 1:**
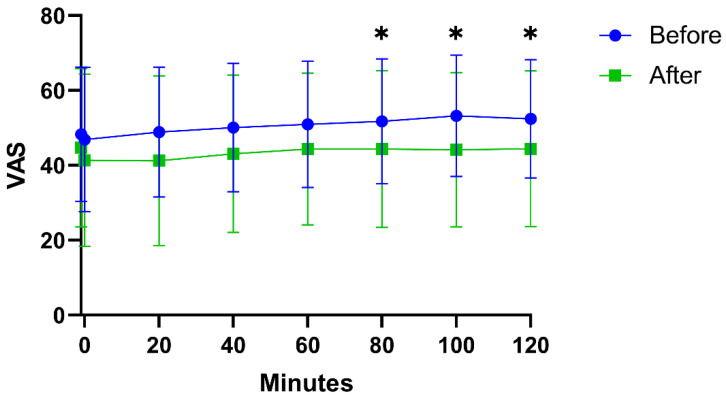
Pain intensity (VAS 0–100) during microdialysis in the vastus lateralis muscle in fibromyalgia patients before and after 15 weeks of resistance exercise intervention. There were significant (*) decrease in VAS at 80, 100, and 120 min after intervention.

Before the start of the intervention, the FM group reported significantly higher scores than CON on pain intensity, HADS, PCS, FIQ, and PDI, which overall indicated greater psychological distress and disability (Table 2). The significance remained post-exercise (Table 2). The FM group showed significant improvements in pain intensity, general health (psychological component), and pain disability after 15 weeks of resistance exercise (Table 2). All subjects rated their pain every 20 min during the microdialysis experiment. There was a significant decrease in pain intensity in the vastus lateralis muscle at time points 80, 100, and 120 min after 15 weeks of resistance exercise in FM compared with the baseline (Figure 1).

As previously has been reported, there were significant differences in the functional tests between FM and CON before the intervention. In the FM group, several strength variables were normalized after exercise (Supplementary Table S1).

### 3.3. Protein Patterns Differentiating FM and CON

**Figure 2:**
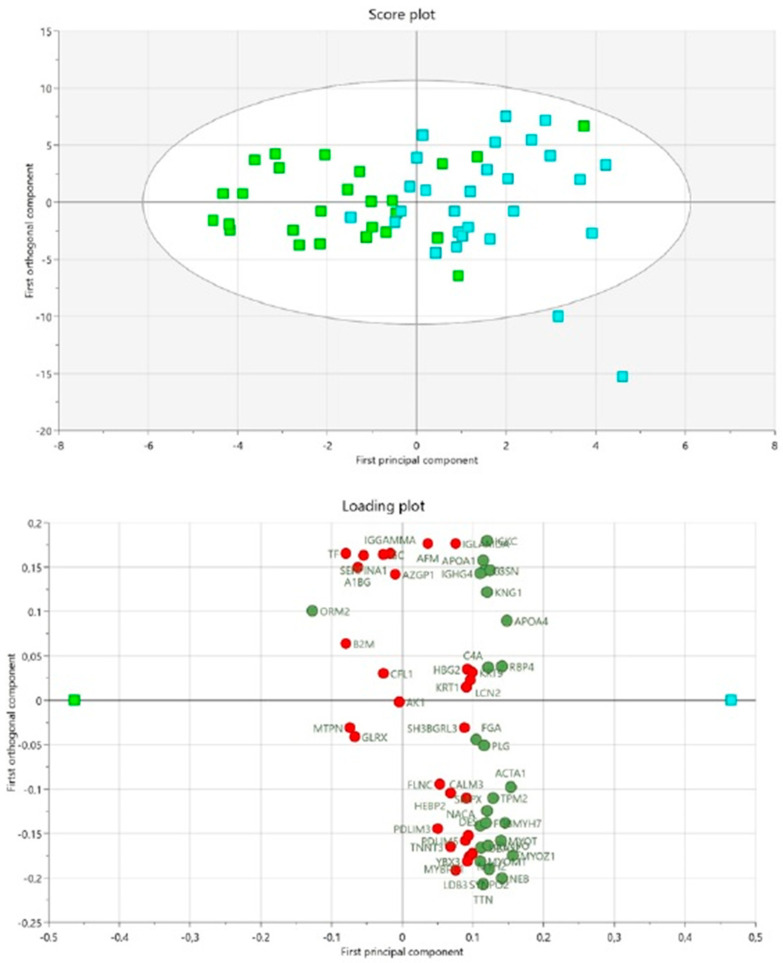
Differences in protein expression between patients with fibromyalgia and healthy controls at baseline using OPLS-DA. The score plot shows each observation and the separation between the fibromyalgia (FM) and healthy control (CON) groups. Green squares = FM group, turquoise squares = CON group. The loading plot shows significant proteins with a VIPpred > 1.0 (green circles) and non-significant proteins VIPpred < 1.0 (red circles). Green square = FM group, turquoise square = CON group. Significant proteins, including abbreviations, are shown in Table 2.

In total, 176 proteins were detected in the interstitial fluid of vastus lateralis using mass spectrometry. There were no outliers that could be detected using the PCA score plot in combination with Hotelling’s T2 test. A significant OPLS-DA model (one predictive and one orthogonal component, R^2^ = 0.45, Q^2^ = 0.19, CV-ANOVA = 0.04) was obtained that could discriminate the groups based on the proteins with a VIPpred > 1.0 and p(corr) > 0.30 (Figure 2). The proteins with the highest VIP-values (i.e., VIPpred > 1.35) were muscle proteins (Myozenin-1, Actin, Myosin-7, Nebulin, Myotilin), and the anti-inflammatory protein Apolipoprotein A-IV, and the inflammatory protein Retinol-binding protein 4 (Table 3). To explore protein changes between FM and CON post-exercise, an OPLS-DA model was created. The model was not significant (CV-ANOVA *p*-value = 0.22, R^2^ = 0.20, Q^2^ = 0.06). 

### 3.4. Pathway Analysis of Proteins at Baseline

**Figure 3:**
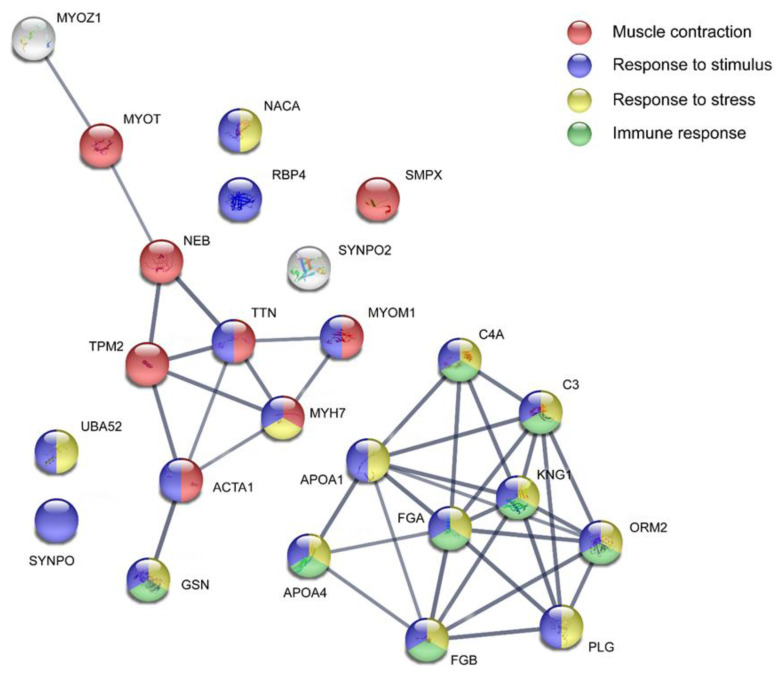
Pathway analysis of protein expression between fibromyalgia patients and healthy controls at baseline. The STRING version 11.0 was used to create the protein interaction analysis (https://string-db.org/ (accessed on 22 June 2022)). The proteins are coded with their protein accession number. The colored nodes represent biological processes. The non-colored nodes were not included in any of the processes marked on this network. For a complete list of biological processes, see Supplementary Table S2. ACTA1: Actin, alpha skeletal muscle; APOA1: Apolipoprotein A-I; APOA4: Apolipoprotein A-IV; C3: Complement C3; C4A: Complement C4-A; FGA: Fibrinogen alpha chain; FGB: Fibrinogen beta chain; GSN: Gelsolin; KNG1: Kininogen-1; MYH7: Myosin-7; MYOM1: Myomesin-1; MYOT: Myotilin; MYOZ1: Myozenin-1; NACA: Nascent polypeptide-associated complex subunit alpha, muscle-specific form; NEB: Nebulin; ORM2: Alpha-1-acid glycoprotein 2; PLG: Plasminogen; RBP4: Retinol-binding protein 4; SMPX: Small muscular protein; SYNPO: Synaptopodin; SYNPO2: Synaptopodin-2; TPM2: Tropomyosin beta chain; TTN: Titin; UBA52: Ubiquitin-60S ribosomal protein L40.

To investigate the biological processes that the proteins were involved in, a protein network analysis using STRING was created based on the 26 significant proteins from the OPLS-DA model between the FM and CON. Immunoglobulin kappa constant and immunoglobulin heavy constant gamma 4 were not identified by the search engine in STRING. A significantly enriched protein network (PPI enrichment *p*-value = 7.13 × 10^−14^) was found. The identified proteins were involved in several biological processes; response to stimulus (FDR = 0.0042), response to stress (FDR = 0.00057), muscle contraction (FDR = 1.91 × 10^−7^), and various processes related to immune response (FDR = 0.0034). For a complete list of identified biological processes, see Supplementary Table S2. Two separate clusters were noted in the network, both containing nine proteins each (Figure 3).

### 3.5. Normalization of Protein Expression Levels between Groups

**Figure 4:**
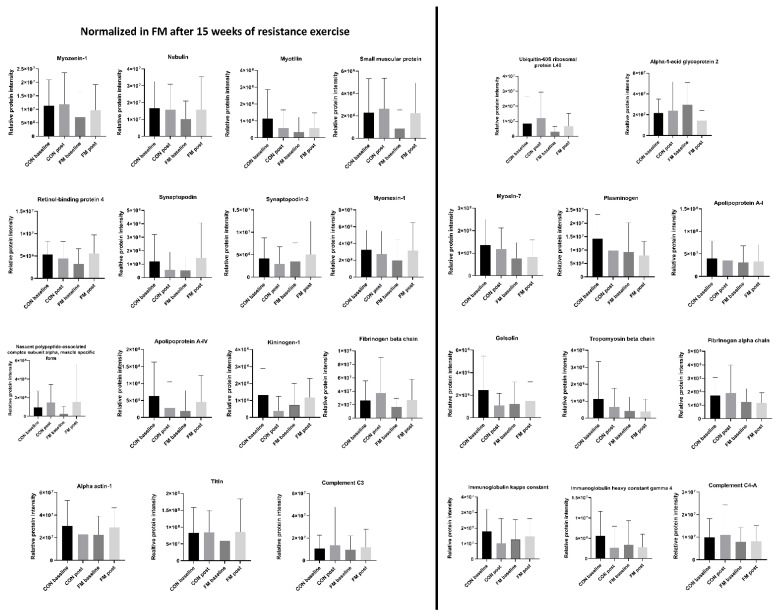
Normalization of proteins post-exercise. Altered relative protein expression levels (mean ± SD) for the 26 significant proteins that were able to discriminate the fibromyalgia (FM) and control (CON) group at baseline. Fifteen proteins were normalized in FM post-exercise.

To further elucidate the potential effect of exercise on the 26 significant proteins that were able to separate FM and CON before the start of the intervention, the protein expression level was compared within the groups post-exercise (Figure 4).

The protein expression level for FM was considered normalized if it reached the same level as the level in CON. Out of the 26 proteins, 15 proteins were normalized in FM. These proteins were myozenin-1, nebulin, myotilin, small muscular protein, retinol-binding protein 4, synaptopodin, synaptopodin-2, myomesin-1, nascent polypeptide-associated complex subunit alpha, apolipoprotein A-IV, kininogen-1, fibrinogen beta chain, Alpha-actin-1, titin, and complement C3.

In the FM group, two proteins were significantly altered post-exercise; Alpha-1-acid glycoprotein was significantly decreased, and retinol-binding protein 4 was significantly increased. In the CON group, four proteins (Immunoglobulin kappa constant, Kininogen-1, Plasminogen, and Immunoglobulin heavy constant gamma 4) differed significantly after the exercise program, where all proteins significantly decreased; for more details see Table 3.

## 4. Discussion

The major findings in this study were (I) the trauma and nociceptive reactions in vastus lateralis in FM patients showed 26 proteins involved in processes related to stress, muscle contractions, external stimuli, and the immune response whose expression levels differed compared to healthy muscle and (II) 15 weeks of resistance exercise had a positive effect on the altered expression levels of proteins caused by the tissue trauma in muscle in FM patients.

This study found altered levels in 26 proteins in muscle interstitial fluid after acute trauma and nociception in FM compared with CON. Furthermore, several of the proteins were muscle proteins, immunity proteins, and proteins involved in coagulation and transporting proteins. The expression level of almost all proteins was lower in the FM group compared with the CON. The only protein with a higher relative expression level in the FM group compared with CON was alpha-1-acid glycoprotein 2.

Studies in FM patients have found an abnormality in muscle metabolism and inflammation [14,15,16,45]. The findings in this study show impairment in stress, muscle contraction, and immune response to acute tissue trauma in FM patients suggesting an ongoing peripheral dysfunction that might maintain the abnormality that has been found in muscle metabolism and inflammation in FM.

The differences in the muscle interstitial proteome between FM and CON after exercise were analyzed using OPLS-DA. No significant (CV-ANOVA > 0.05) model could be found, indicating that there are no significant differences in the expression level of proteins in FM compared to CON. Targeted data analysis against the 26 significant proteins that were decreased in FM at baseline showed increased levels of all proteins after the intervention. The levels of the majority of the proteins were increased to the levels of the healthy controls; however, the levels of only two proteins were statistically significantly increased after the intervention compared with the baseline. The findings indicated that the exercise intervention had an impact on protein normalization in the vastus lateralis in FM patients. The identified protein pathways in this study are in accordance with previously identified pathways using biological fluids such as plasma/serum, saliva, and cerebrospinal fluid in FM patients [18,30,46,47,48,49]. Most studies have focused on the plasma proteome in FM, which represents studies of both peripheral tissue and systemic changes since the blood is in contact will all tissue. Recently, Han et al. found upregulated proteins involved in inflammation and coagulation when investigating the serum proteome in patients with FM [46]. Another FM study of the serum proteome by Ramirez-Tejero et al. found upregulated proteins involved in the coagulation cascade, complement system, and proteins involved in the acute-phase response [47]. In this cohort of FM and CON, we have previously identified systemic differences in proteins involved in immunity, blood coagulation, metabolic, and inflammatory processes when investigating the plasma proteome at baseline [30]. Additional analysis of the plasma proteome has revealed normalization of plasma proteins after 15 weeks of exercise, involved in muscle structure development, metabolic processes, immunity, and stress response [31]. Interestingly, even though these studies are based on serum/plasma proteome, similar processes were found in our present study focusing on acute nociception in the vastus lateralis muscle during the trauma phase. Our study showed decreased levels of protein expression in FM at baseline compared with CON, and after 15 weeks of resistance exercise, several of these proteins were normalized, reaching similar levels as the control. The present study and a recent one [31] thus indicate that exercise alters proteins both systemically and locally in the vastus lateralis muscle, especially proteins that seem to be involved in the important processes of the immune system and inflammation, e.g., kininogen-1, fibrinogen beta chain, apolipoprotein A-IV, complement C3, and alpha-1 acid glycoprotein. Hence, further studies to confirm these findings are warranted.

Currently, studies on proteome profiling in muscles using microdialysis on FM patients are limited. Earlier microdialysis studies have reported the identification of other substances. Ernberg et al. have previously investigated the pro-inflammatory cytokine profile in the interstitial fluid from the same cohort. No effect on the pro-inflammatory cytokines TNF, Interleukin (IL)-1beta, IL-8, or IL-6 was found after 15 weeks of resistance exercise between FM and control [28]. An earlier study reported higher levels of serotonin in the masseter muscle of the FM group in the trauma phase but not during the steady state [24]. Further, altered levels of metabolites for the period 20–40 min after catheter insertion in a microdialysis study investigating biomarkers after acute tissue trauma were found in both patients with chronic musculoskeletal pain and healthy controls [17]. The metabolites investigated in that report were glucose, lactate, pyruvate, and glycerol. Gerdle et al. found increased interstitial concentrations of lactate, glutamate, and pyruvate in patients with FM compared with healthy controls [15]. The increased concentrations of glutamate and pyruvate did, however, decrease after a 15-week exercise intervention. Moreover, in this present study, we found numerous expressed muscle proteins, which can be explained by muscle damage during microdialysis [24]. Most of the proteins were also involved in processes related to a stimulus, which could reflect the insertion of a microdialysis catheter and acute nociception in vastus lateralis. The relative protein expression level was lower at baseline in the FM group compared with CON. This finding might suggest inhibition of the acute inflammatory response, which has been reported to play an important role in the prolongation of pain [50].

Studying the muscle morphology with biopsy in patients with FM has also been a matter of interest. However, the procedure is invasive, and studies have not reported specific findings that can contribute to the diagnosis of FM [51,52]. Hence, Olausson et al. have studied the trapezius muscle proteome from women with chronic widespread pain (CWP; the majority fulfilled the FM diagnosis) and the interstitial fluid from women with trapezius myalgia and CWP compared with healthy controls [25,53]. These studies have shown alteration in enzymatic proteins involved in several metabolic processes, such as gluconeogenesis and glycolysis, and found proteins related to nociceptive processes of inflammation, muscle damage, recovery, and stress [25,53].

Furthermore, other studies have found differences between patients with FM and healthy controls regarding muscle fiber dysfunction due to oxidative damage, mitochondrial abnormalities, vasomotor dysregulation, chronic inflammation, and defects in capillary microcirculation [16,54,55]. Therefore, the outcome of the present study cannot be regarded as unexpected, as the muscles in FM patients react differently than in healthy controls during acute nociception. From our obtained results and previously reported studies, it is known that exercise improves both muscle strength and pain intensity [10,13,34]. Holloway and colleagues have studied the proteome of vastus lateralis in healthy men after interval exercise training [56]. Several of the proteins that were altered in these healthy controls post-exercise was myofibrillar proteins. This is in line with the changes of similar proteins (e.g., tropomyosin beta chain, myosin −1, and −7) in our study. However, in our controls, the protein levels were decreased, and in FM, the relative protein expression levels were increased. The reason for discrepancies in protein expression levels could be due to the different proteome studied, exercise intervention, men versus women, and disease outcomes. Thus, the increase in the relative protein abundance of observed proteins indicates that there has been a change in the vastus lateralis muscle in the FM patients after 15 weeks of resistance exercise. As previously mentioned, to study the proteins and their levels further, we would have to analyze the dialysate samples after the trauma phase. The reason for this is to study how the protein levels differ after 120 min when the tissue is more normalized compared with during acute nociception.

One of the main strengths of this study is the well-characterized material that describes the study population. It is also the first proteomic investigation of changes in the interstitial muscle proteome after 15 weeks of resistance exercise in FM compared with healthy controls. The limitations of this study should also be addressed. First, the BMI differed significantly between the two groups, but due to the low number of samples (obesity *n* = 5, high BMI *n* = 6 and normal BMI *n* = 10) no statistical analysis between the different BMI group was performed. In future studies a subset of FM patients with low and high BMIs should be included to investigate the effect of BMI on muscle interstitial proteome and acute nociception. Second, even though the number of participants in this study was sufficient, an increased number of participants should be considered in future studies. Third, as mentioned by Christidis et al. [27], since patients with FM were recruited to an exercise study, they most likely were less affected by their disorder compared with FM patients in general. Therefore, by including patients with a more severe form of FM, the outcome of the result might be different. Fourth, this study was solely performed on women, thus, the results are not generalizable for everyone with FM. Finally, antibody validation of the results should be used for a more accurate verification of the proteins, however this was not executed in this study.

In conclusion, for the first time the proteome of interstitial fluid from vastus lateralis in FM after an acute tissue trauma was explored. This study showed that 15 weeks of resistance exercise improved the reaction of the muscle after an acute nociception in FM patients. Microdialysis was a good experimental model to study peripheral changes of proteins and acute nociception in patients with chronic musculoskeletal pain. Physical function was improved in FM and the levels of several proteins involved in muscle contraction, stress, and immune response were normalized after 15 weeks of exercise intervention. This study shows promising results for exercise in this patient group and the effect of exercise modulating peripheral muscle proteins in patients with FM. Future study comparing FM with other chronic pain states are warranted to investigate the specificity of the identified protein biomarkers of chronic pain mechanisms in fibromyalgia.

## Figures and Tables

**Table 1:** Baseline characteristics of healthy controls (CON) and fibromyalgia patients (FM). The data are presented as mean and standard deviation (±SD). BMI: body mass index; FM = fibromyalgia; BP: blood pressure.

Variables	CON *n* = 28	FM *n* = 26	*p*-Value
Age (years)	53.4 (±9.8)	54.0 (±8.1)	0.98
BMI (kg/m^2^)	24.5 (±4.8)	27.5 (±5.4)	0.02
FM duration (years)	-	14.1 (±7.8)	-
Systolic BP (mmHg)	131 (±15.4)	134 (±20)	0.73
Diastolic BP (mmHg)	82 (±8.2)	84 (±9.9)	0.59
Tender points (*n*)	-	16 (±1.5)	-

**Table 2:** Questionnaire data on pain intensity by the Visual Analog Scale (Global VAS), two subscales (anxiety and depression) of the Hospital Anxiety and Depression Scales (HADS), Pain Catastrophizing Scale (PCS), Fibromyalgia Impact Questionnaire (FIQ), Short Form Health Survey 36 (SF-36) with Physical Summary Component measure (PSC) and Mental Component Summary measure (MCS), and Pain Disability Index (PDI). Data are presented as mean and standard deviation (± SD). FM = fibromyalgia, CON = healthy controls.

Clinical Variables	CON Baseline *n* = 28	FM Baseline *n* = 26	FM vs. CON Baseline *p*-Value	CON Post Exercise *n* = 26	FM Post Exercise *n* = 25	FM vs. CON Post Exercise *p*-Value	FM Baseline vs. Post Exercise *p*-Value
Pain intensity (Global VAS)	2.2 (±6.4)	48.4 (±24.0)	<0.001	5.5 (±15.6)	34.1 (±23.3)	<0.001	0.021
HADS depression	1.6 (±1.7)	6.0 (±4.0)	<0.001	1.5 (±2.1)	5.5 (±4.3)	<0.001	0.138
HADS anxiety	3.3 (±3.1)	6.7 (±4.4)	0.003	2.8 (±3.0)	6.8 (±4.7)	0.001	1.00
PCS	7.0 (±11.5)	17.8 (±11.4)	0.002	3.5 (±4.8)	16.7 (±10.1)	<0.001	0.244
FIQ	6.9 (±9.3)	59. 8 (±13.9)	<0.001	9.1 (±13.9)	55.2 (±20.1)	<0.001	0.179
SF-36 PSC	54. 6 (±4.6)	29.6 (±7.7)	<0.001	53.3 (±9.2)	33.1 (±8.6)	<0.001	0.036
SF-36 MCS	51.4 (±6.0)	42.7 (±10.9)	0.010	52.4 (±7.4)	43.3 (±11.9)	0.003	0.274
PDI	8.5 (±2.9)	36.2 (±13.7)	<0.001	10.1 (±6.9)	31.5 (±12.9)	<0.001	0.015

**Table 3:** Summary of the Uniprot accession number protein name, VIP predictive, p(corr), mean value and standard deviation (±SD) of protein intensity, *p*-value (within groups), the ratio within and between groups before and after 15-week exercise intervention exercise. The accession numbers are according to the UniProt database. The proteins correspond to visualized proteins in the loading plot of Figure 2 and are sorted depending on their VIPpred value. The higher VIPpred, the more important regressor for the model. p(corr) is the multivariate correlation coefficient of each variable (protein) for the model. Protein abbreviation (protein abbrev.) corresponds to significant proteins from loading plot Figure 2. Significant proteins within groups are marked in bold.

Protein Accession Number (Uniprot)	Protein Abbrev.	Protein Name	VIPpred	p(corr)	CON Baseline Mean (±SD) *n* = 28	CON Post Mean (±SD) *n* = 26	*p*-Value CON Baseline vs. Post	Ratio CON Post/Baseline	FM Baseline Mean (±SD) *n* = 26	FM Post Mean (±SD) *n* = 25	*p*-Value FM Baseline vs. Post	Ratio FM Post/Baseline	Ratio FM/CON Baseline	Ratio FM/CON Post
Q9NP98	MYOZ1	Myozenin-1	1.54	0.50	1.15 × 10^7^ (±9.19 × 10^6^)	1.16 × 10^7^ (±1.13 × 10^7^)	0.79	1.00	6.19 × 10^6^ (±8.65 × 10^6^)	8.87 × 10^6^ (±8.99 × 10^6^)	0.26	1.43	0.54	0.77
P68133	ACTA1	Actin, alpha skeletal muscle (Alpha-actin-1)	1.52	0.49	2.96 × 10^7^ (±2.11 × 10^7^)	2.47 × 10^7^ (±1.43 × 10^7^)	0.07	0.84	2.11 × 10^7^ (±1.56 × 10^7^)	2.72 × 10^7^ (±1.70 × 10^7^)	0.14	1.29	0.71	1.10
P06727	APOA4	Apolipoprotein A-IV	1.45	0.47	5.86 × 10^5^ (±9.64 × 10^5^)	2.46 × 10^5^ (±7.31 × 10^5^)	0.24	0.42	2.29 × 10^5^ (±6.56 × 10^5^)	3.83 × 10^5^ (±7.31 × 10^5^)	0.12	1.67	0.39	1.56
P12883	MYH7	Myosin-7	1.43	0.46	1.33 × 10^8^ (±1.04 × 10^8^)	1.18 × 10^8^ (±8.98 × 10^7^)	0.31	0.89	6.74 × 10^7^ (±6.70 × 10^7^)	7.48 × 10^7^ (±7.31 × 10^7^)	0.73	1.11	0.51	0.63
P20929	NEB	Nebulin	1.39	0.45	1.72 × 10^7^ (±1.70 × 10^7^)	1.72 × 10^7^ (±1.71 × 10^7^)	0.76	1.00	9.44 × 10^6^ (±1.01 × 10^7^)	1.46 × 10^7^ (±1.86 × 10^7^)	0.55	1.55	0.55	0.85
P02753	RBP4	Retinol-binding protein 4	1.38	0.45	5.23 × 10^6^ (±2.86 × 10^6^)	4.19 × 10^6^ (±3.78 × 10^6^)	0.26	0.80	3.20 × 10^6^ (±3.14 × 10^6^)	5.59 × 10^6^ (±4.03 × 10^6^)	**0.03**	1.75	0.61	1.33
Q9UBF9	MYOT	Myotilin	1.37	0.44	1.16 × 10^6^ (±1.72 × 10^6^)	7.62 × 10^5^ (±1.23 × 10^6^)	0.24	0.65	3.08 × 10^5^ (±8.26 × 10^5^)	6.27 × 10^5^ (±8.88 × 10^5^)	0.26	2.04	0.26	0.82
P07951	TPM2	Tropomyosin beta chain	1.26	0.41	1.18 × 10^6^ (±2.23 × 10^6^)	7.06 × 10^5^ (±1.15 × 10^6^)	0.21	0.60	3.95 × 10^5^ (±8.04 × 10^5^)	4.36 × 10^5^ (±8.28 × 10^5^)	0.78	1.10	0.34	0.62
P19652	ORM2	Alpha-1-acid glycoprotein 2	1.25	−0.40	2.08 × 10^7^ (±1.37 × 10^7^)	2.29 × 10^7^ (±2.62 × 10^7^)	0.58	1.10	2.98 × 10^7^ (±2.02 × 10^7^)	1.55 × 10^7^ (±1.03 × 10^7^)	**0.01**	0.52	1.44	0.68
P06396	GSN	Gelsolin	1.23	0.40	2.64 × 10^6^ (±2.87 × 10^6^)	9.78 × 10^5^ (±1.06 × 10^6^)	0.07	0.37	1.38 × 10^6^ (±2.03 × 10^6^)	1.66 × 10^6^ (±1.67 × 10^6^)	0.44	1.20	0.52	1.69
Q9UMS6	SYNPO2	Synaptopodin-2	1.21	0.39	4.60 × 10^−6^ (±5.05 × 10^6^)	3.49 × 10^6^ (±4.50 × 10^6^)	0.45	0.76	2.94 × 10^6^ (±3.93 × 10^6^)	4.87 × 10^6^ (±7.05 × 10^6^)	0.84	1.66	0.64	1.40
P0C0L4	C4A	Complement C4-A	1.19	0.39	9.33 × 10^6^ (±7.61 × 10^6^)	1.03 × 10^7^ (±1.27 × 10^7^)	0.63	1.11	7.60 × 10^6^ (±5.98 × 10^6^)	8.48 × 10^6^ (±6.77 × 10^6^)	0.93	1.12	0.81	0.82
Q8N3V7	SYNPO	Synaptopodin	1.19	0.39	1.31 × 10^6^ (±2.04 × 10^6^)	6.77 × 10^5^ (±1.46 × 10^6^)	0.17	0.52	4.79 × 10^5^ (±9.18 × 10^5^)	1.35 × 10^6^ (±2.46 × 10^6^)	0.14	2.83	0.37	2.00
P01834	IGKC	Immunoglobulin kappa constant	1.18	0.38	1.83 × 10^7^ (±1.37 × 10^7^)	9.30 × 10^6^ (±1.51 × 10^7^)	**0.02**	0.51	1.28 × 10^7^ (±1.14 × 10^7^)	1.49 × 10^7^ (±1.25 × 10^7^)	0.35	1.16	0.70	1.60
P01042	KNG1	Kininogen-1	1.18	0.38	1.43 × 10^6^ (±1.55 × 10^6^)	3.29 × 10^5^ (±8.31 × 10^5^)	**0.04**	0.23	8.79 × 10^5^ (±1.31 × 10^6^)	1.19 × 10^6^ (±1.17 × 10^6^)	0.16	1.36	0.62	3.62
Q9UHP9	SMPX	Small muscular protein	1.18	0.38	2.48 × 10^6^ (±3.23 × 10^6^)	2.91 × 10^6^ (±3.56 × 10^6^)	0.30	1.17	1.01 × 10^6^ (±1.72 × 10^6^)	2.16 × 10^6^ (±2.60 × 10^6^)	0.12	2.14	0.41	0.74
P02675	FGB	Fibrinogen beta chain	1.17	0.38	2.50 × 10^7^ (±2.75 × 10^7^)	3.50 × 10^7^ (±5.03 × 10^7^)	0.90	1.40	1.52 × 10^7^ (±1.36 × 10^7^)	2.44 × 10^7^ (±2.91 × 10^7^)	0.32	1.61	0.61	0.70
P01024	C3	Complement C3	1.15	0.37	1.14 × 10^7^ (±1.16 × 10^7^)	1.21 × 10^7^ (±3.24 × 10^7^)	0.13	1.07	9.14 × 10^6^ (±1.17 × 10^7^)	1.21 × 10^7^ (±1.57 × 10^7^)	0.60	1.33	0.80	1.00
P00747	PLG	Plasminogen	1.14	0.37	1.32 × 10^7^ (±8.55 × 10^6^)	9.35 × 10^6^ (±6.78 × 10^6^)	**0.03**	0.71	8.33 × 10^6^ (±1.02 × 10^7^)	8.06 × 10^6^ (±4.82 × 10^6^)	0.71	0.97	0.63	0.86
P02647	APOA1	Apolipoprotein A-I	1.13	0.37	4.25 × 10^7^ (±3.80 × 10^7^)	3.20 × 10^7^ (±8.00 × 10^7^)	0.06	0.75	2.98 × 10^7^ (±3.35 × 10^7^)	3.15 × 10^7^ (±3.56 × 10^7^)	0.91	1.05	0.70	0.98
Q8WZ42	TTN	Titin	1.13	0.37	8.73 × 10^7^ (±8.22 × 10^7^)	9.42 × 10^7^ (±8.56 × 10^7^)	0.83	1.08	5.49 × 10^7^ (±4.46 × 10^7^)	8.13 × 10^7^ (±9.36 × 10^7^)	0.45	1.48	0.63	0.86
P52179	MYOM1	Myomesin-1	1.09	0.35	3.25 × 10^6^ (±2.34 × 10^6^)	3.04 × 10^6^ (±3.09 × 10^6^)	0.19	0.94	1.84 × 10^6^ (±2.33 × 10^6^)	2.86 × 10^6^ (±3.19 × 10^6^)	0.17	1.55	0.57	0.94
E9PAV3	NACA	Nascent polypeptide-associated complex subunit alpha, muscle-specific form	1.09	0.35	9.37 × 10^5^ (±1.67 × 10^6^)	1.74 × 10^6^ (±2.26 × 10^6^)	0.39	1.86	2.23 × 10^5^ (±7.01 × 10^5^)	1.41 × 10^6^ (±3.74 × 10^6^)	0.16	6.31	0.24	0.81
P62987	UBA52	Ubiquitin-60S ribosomal protein L40	1.09	0.35	9.94 × 10^6^ (±2.10 × 10^7^)	1.15 × 10^7^ (±1.65 × 10^7^)	0.34	1.16	2.91 × 10^6^ (±3.32 × 10^6^)	6.63 × 10^6^ (±8.65 × 10^6^)	0.13	2.28	0.29	0.57
P01861	IGHG4	Immunoglobulin heavy constant gamma 4	1.08	0.35	6.01 × 10^6^ (±5.90 × 10^6^)	2.39 × 10^6^ (±5.04 × 10^6^)	**0.04**	0.40	3.46 × 10^6^ (±5.57 × 10^6^)	2.92 × 10^6^ (±3.51 × 10^6^)	0.92	0.85	0.58	1.22
P02671	FGA	Fibrinogen alpha chain	1.03	0.33	1.59 × 10^8^ (±1.25 × 10^8^)	1.84 × 10^8^ (±1.95 × 10^8^)	0.85	1.15	1.20 × 10^8^ (±9.54 × 10^7^)	1.12 × 10^8^ (±7.09 × 10^7^)	0.82	0.93	0.75	0.61

## Data Availability

The datasets generated in this study are not publicly available as the Ethical Review Board has not approved the public availability of these data.

## Supplementary Materials

The following supporting information can be downloaded at: https://www.mdpi.com/article/10.3390/biomedicines11010206/s1, Table S1: Measurement of physical capacity and functional tests of muscle strength, and pressure pain threshold before and after 15 weeks of resistance exercise in fibromyalgia patients and healthy controls; Table S2: Identified biological processes.

## References

[1] Gatchel R.J., Peng Y.B., Peters M.L., Fuchs P.N., Turk D.C. (2007). The biopsychosocial approach to chronic pain: Scientific advances and future directions. Psychol. Bull.

[2] Perez de Heredia-Torres M., Huertas-Hoyas E., Máximo-Bocanegra N., Palacios-Ceña D., Fernández-De-Las-Peñas C. (2016). Cognitive performance in women with fibromyalgia: A case-control study. Aust. Occup. Ther. J..

[3] Siracusa R., Paola R.D., Cuzzocrea S., Impellizzeri D. (2021). Fibromyalgia: Pathogenesis, Mechanisms, Diagnosis and Treatment Options Update. Int. J. Mol. Sci..

[4] Terol Cantero M.C., Buunk A.P., Cabrera V., Bernabé M., Martin-Aragón Gelabert M. (2020). Profiles of Women with Fibromyalgia and Social Comparison Processes. Front. Psychol..

[5] Cetingok S., Seker O., Cetingok H. (2022). The relationship between fibromyalgia and depression, anxiety, anxiety sensitivity, fear avoidance beliefs, and quality of life in female patients. Medicine.

[6] Clauw D.J. (2014). Fibromyalgia: A clinical review. JAMA.

[7] Wolfe F., Clauw D.J., Fitzcharles M.A., Goldenberg D.L., Häuser W., Katz R.L., Mease P.J., Russell A.S., Russell I.J., Walitt B. (2016). 2016 Revisions to the 2010/2011 fibromyalgia diagnostic criteria. Semin. Arthritis. Rheum..

[8] Marques A.P., Santo A.S.D.E., Berssaneti A.A., Matsutani L.A., Yuan S.L.K. (2017). Prevalence of fibromyalgia: Literature review update. Rev. Bras. De Reumatol. (Engl. Ed.).

[9] Macfarlane G.J., Kronisch C., Dean L.E., Atzeni F., Häuser W., Fluß E., Choy E., Kosek E., Amris K., Branco J. (2017). EULAR revised recommendations for the management of fibromyalgia. Ann. Rheum. Dis..

[10] Busch A.J., Webber S.C., Richards R.S., Bidonde J., Schachter C.L., Schafer L.A., Danyliw A., Sawant A., Dal Bello-Haas V., Rader T. (2013). Resistance exercise training for fibromyalgia. Cochrane Database Syst. Rev..

[11] Assumpção A., Matsutani L.A., Yuan S.L., Santo A.S., Sauer J., Mango P., Marques A.P. (2018). Muscle stretching exercises and resistance training in fibromyalgia: Which is better? A three-arm randomized controlled trial. Eur. J. Phys. Rehabil. Med..

[12] Ericsson A., Palstam A., Larsson A., Löfgren M., Bileviciute-Ljungar I., Bjersing J., Gerdle B., Kosek E., Mannerkorpi K. (2016). Resistance exercise improves physical fatigue in women with fibromyalgia: A randomized controlled trial. Arthritis Res. Ther..

[13] Hooten M.W., Qu W., Townsend C.O., Judd J.W. (2012). Effects of strength vs aerobic exercise on pain severity in adults with fibromyalgia: A randomized equivalence trial. Pain.

[14] Gerdle B., Larsson B., Forsberg F., Ghafouri N., Karlsson L., Stensson N., Ghafouri B. (2014). Chronic widespread pain: Increased glutamate and lactate concentrations in the trapezius muscle and plasma. Clin. J. Pain.

[15] Gerdle B., Söderberg K., Salvador Puigvert L., Rosendal L., Larsson B. (2010). Increased interstitial concentrations of pyruvate and lactate in the trapezius muscle of patients with fibromyalgia: A microdialysis study. J. Rehabil. Med..

[16] Gerdle B., Ghafouri B., Lund E., Bengtsson A., Lundberg P., Ettinger-Veenstra H.V., Leinhard O.D., Forsgren M.F. (2020). Evidence of Mitochondrial Dysfunction in Fibromyalgia: Deviating Muscle Energy Metabolism Detected Using Microdialysis and Magnetic Resonance. J. Clin. Med..

[17] Sorensen L.B., Gazerani P., Wåhlén K., Ghafouri N., Gerdle B., Ghafouri B. (2018). Investigation of biomarkers alterations after an acute tissue trauma in human trapezius muscle, using microdialysis. Sci. Rep..

[18] Kadetoff D., Lampa J., Westman M., Andersson M., Kosek E. (2012). Evidence of central inflammation in fibromyalgia-increased cerebrospinal fluid interleukin-8 levels. J. Neuroimmunol..

[19] O’Mahony L.F., Srivastava A., Mehta P., Ciurtin C. (2021). Is fibromyalgia associated with a unique cytokine profile? A systematic review and meta-analysis. Rheumatology.

[20] Bjersing J.L., Dehlin M., Erlandsson M., Bokarewa M.I., Mannerkorpi K. (2012). Changes in pain and insulin-like growth factor 1 in fibromyalgia during exercise: The involvement of cerebrospinal inflammatory factors and neuropeptides. Arthritis Res. Ther..

[21] Jablochkova A., Bäckryd E., Kosek E., Mannerkorpi K., Ernberg M., Gerdle B., Ghafouri B. (2019). Unaltered low nerve growth factor and high brain-derived neurotrophic factor levels in plasma from patients with fibromyalgia after a 15-week progressive resistance exercise. J. Rehabil. Med..

[22] D’Amico R., Fusco R., Siracusa R., Impellizzeri D., Peritore A.F., Gugliandolo E., Interdonato L., Sforza A.M., Crupi R., Cuzzocrea S. (2021). Inhibition of P2X7 Purinergic Receptor Ameliorates Fibromyalgia Syndrome by Suppressing NLRP3 Pathway. Int. J. Mol. Sci..

[23] Shippenberg T.S., Thompson A.C. (1997). Overview of microdialysis. Curr. Protoc. Neurosci..

[24] Ernberg M., Hedenberg-Magnusson B., Alstergren P., Kopp S. (1999). The level of serotonin in the superficial masseter muscle in relation to local pain and allodynia. Life Sci..

[25] Turkina M.V., Ghafouri N., Gerdle B., Ghafouri B. (2017). Evaluation of dynamic changes in interstitial fluid proteome following microdialysis probe insertion trauma in trapezius muscle of healthy women. Sci. Rep..

[26] Olausson P., Gerdle B., Ghafouri N., Sjöström D., Blixt E., Ghafouri B. (2015). Protein alterations in women with chronic widespread pain—An explorative proteomic study of the trapezius muscle. Sci. Rep..

[27] Christidis N., Ghafouri B., Larsson A., Palstam A., Mannerkorpi K., Bileviciute-Ljungar I., Löfgren M., Bjersing J., Kosek E., Gerdle B. (2015). Comparison of the Levels of Pro-Inflammatory Cytokines Released in the Vastus Lateralis Muscle of Patients with Fibromyalgia and Healthy Controls during Contractions of the Quadriceps Muscle—A Microdialysis Study. PLoS ONE.

[28] Ernberg M., Christidis N., Ghafouri B., Bileviciute-Ljungar I., Löfgren M., Larsson A., Palstam A., Bjersing J., Mannerkorpi K., Kosek E. (2016). Effects of 15 weeks of resistance exercise on pro-inflammatory cytokine levels in the vastus lateralis muscle of patients with fibromyalgia. Arthritis Res. Ther..

[29] Gerdle B., Ernberg M., Mannerkorpi K., Larsson B., Kosek E., Christidis N., Ghafouri B. (2016). Increased Interstitial Concentrations of Glutamate and Pyruvate in Vastus Lateralis of Women with Fibromyalgia Syndrome Are Normalized after an Exercise Intervention—A Case-Control Study. PLoS ONE.

[30] Wåhlén K., Ernberg M., Kosek E., Mannerkorpi K., Gerdle B., Ghafouri B. (2020). Significant correlation between plasma proteome profile and pain intensity, sensitivity, and psychological distress in women with fibromyalgia. Sci. Rep..

[31] Wåhlén K., Yan H., Welinder C., Ernberg M., Kosek E., Mannerkorpi K., Gerdle B., Ghafouri B. (2022). Proteomic Investigation in Plasma from Women with Fibromyalgia in Response to a 15-wk Resistance Exercise Intervention. Med. Sci. Sport. Exerc..

[32] Palstam A., Larsson A., Bjersing J., Löfgren M., Ernberg M., Bileviciute-Ljungar I., Ghafouri B., Sjörs A., Larsson B., Gerdle B. (2014). Perceived exertion at work in women with fibromyalgia: Explanatory factors and comparison with healthy women. J. Rehabil. Med..

[33] Wolfe F., Smythe H.A., Yunus M.B., Bennett R.M., Bombardier C., Goldenberg D.L., Tugwell P., Campbell S.M., Abeles M., Clark P. (1990). The American College of Rheumatology 1990 Criteria for the Classification of Fibromyalgia. Report of the Multicenter Criteria Committee. Arthritis Rheum.

[34] Larsson A., Palstam A., Löfgren M., Ernberg M., Bjersing J., Bileviciute-Ljungar I., Gerdle B., Kosek E., Mannerkorpi K. (2015). Resistance exercise improves muscle strength, health status and pain intensity in fibromyalgia—A randomized controlled trial. Arthritis Res. Ther..

[35] Zigmond A.S., Snaith R. (1983). The hospital anxiety and depression scale. Acta Psychiatr. Scand..

[36] Boonstra A.M., Schiphorst Preuper H.R., Balk G.A., Stewart R.E. (2014). Cut-off points for mild, moderate, and severe pain on the visual analogue scale for pain in patients with chronic musculoskeletal pain. Pain.

[37] Sullivan M.J.L., Bishop S., Pivik J. (1995). The Pain Catastrophizing Scale: Development and validation. Psychol. Assess..

[38] Hedin P.J., Hamne M., Burckhardt C.S., Engström-Laurent A. (1995). The Fibromyalgia Impact Questionnaire, a Swedish translation of a new tool for evaluation of the fibromyalgia patient. Scand. J. Rheumatol..

[39] Ware J.E., Sherbourne C.D. (1992). The MOS 36-item short-form health survey (SF-36). I. Conceptual framework and item selection. Med. Care.

[40] Ware J.E., Gandek B. (1998). Overview of the SF-36 health survey and the international quality of life assessment (IQOLA) project. J. Clin. Epidemiol..

[41] Wåhlén K., Olausson P., Carlsson A., Ghafouri N., Gerdle B., Ghafouri B. (2017). Systemic alterations in plasma proteins from women with chronic widespread pain compared to healthy controls: A proteomic study. J. Pain Res..

[42] Wheelock A.M., Wheelock C.E. (2013). Trials and tribulations of omics data analysis: Assessing quality of SIMCA-based multivariate models using examples from pulmonary medicine. Mol. Biosyst..

[43] Eriksson L., Johansson E., Kettaneh-Wold N., Trygg J., Wikström C., Wold S. (2016). Multi- and Megavariate Data Analysis, Part I and II.

[44] Szklarczyk D., Gable A.L., Lyon D., Junge A., Wyder S., Huerta-Cepas J., Simonovic M., Doncheva N.T., Morris J.H., Bork P. (2019). STRING v11: Protein-protein association networks with increased coverage, supporting functional discovery in genome-wide experimental datasets. Nucleic Acids Res..

[45] Ghafouri B., Edman E., Löf M., Lund E., Leinhard O.D., Lundberg P., Forsgren M.F., Gerdle B., Dong H.J. (2022). Fibromyalgia in women: Association of inflammatory plasma proteins, muscle blood flow, and metabolism with body mass index and pain characteristics. Pain Rep..

[46] Han C.L., Sheng Y.C., Wang S.Y., Chen Y.H., Kang J.H. (2020). Serum proteome profiles revealed dysregulated proteins and mechanisms associated with fibromyalgia syndrome in women. Sci. Rep..

[47] Ramirez-Tejero J.A., Martínez-Lara E., Rus A., Camacho M.V., Del Moral M.L., Siles E. (2018). Insight into the biological pathways underlying fibromyalgia by a proteomic approach. J. Proteom..

[48] Khoonsari P.E., Ossipova E., Lengqvist J., Svensson C.I., Kosek E., Kadetoff D., Jakobsson P.J., Kultima K., Lampa J. (2019). The human CSF pain proteome. J. Proteom..

[49] Ciregia F., Giacomelli C., Giusti L., Boldrini C., Piga I., Pepe P., Consensi A., Gori S., Lucacchini A., Mazzoni M.R. (2019). Putative salivary biomarkers useful to differentiate patients with fibromyalgia. J. Proteom..

[50] Parisien M., Lima L.V., Dagostino C., El-Hachem N., Drury G.L., Grant A.V., Huising J., Verma V., Meloto C.B., Silva J.R. (2022). Acute inflammatory response via neutrophil activation protects against the development of chronic pain. Sci. Transl. Med..

[51] Schrøder H.D., Drewes A.M., Andreasen A. (1993). Muscle biopsy in fibromyalgia. J. Musculoskelet. Pain.

[52] Ruggiero L., Manganelli F., Santoro L. (2018). Muscle pain syndromes and fibromyalgia: The role of muscle biopsy. Curr. Opin. Support Palliat. Care.

[53] Olausson P., Gerdle B., Ghafouri N., Larsson B., Ghafouri B. (2012). Identification of proteins from interstitium of trapezius muscle in women with chronic myalgia using microdialysis in combination with proteomics. PLoS ONE.

[54] Srikuea R., Symons T.B., Long D.E., Lee J.D., Shang Y., Chomentowski P.J., Yu G., Crofford L.J., Peterson C.A. (2013). Association of fibromyalgia with altered skeletal muscle characteristics which may contribute to postexertional fatigue in postmenopausal women. Arthritis Rheum.

[55] Katz D.L., Greene L., Ali A., Faridi Z. (2007). The pain of fibromyalgia syndrome is due to muscle hypoperfusion induced by regional vasomotor dysregulation. Med. Hypotheses.

[56] Holloway K.V., O’Gorman M., Woods P., Morton J.P., Evans L., Cable N.T., Goldspink D.F., Burniston J.G. (2009). Proteomic investigation of changes in human vastus lateralis muscle in response to interval-exercise training. Proteomics.

