# Supplementary material for: The Vastus Lateralis Muscle Interstitium Proteome Changes after an Acute Nociception in Patients with Fibromyalgia Compared to Healthy Subjects—A Microdialysis Study
Source: Biomedicines. 2023 Jan 13;11(1):206. doi: 10.3390/biomedicines11010206 (PMC9856129; doi:10.3390/biomedicines11010206)
Supplement: Supplementary file 1 [file biomedicines-11-00206-s001.zip › biomedicines-2126678-supplementary.pdf]

Table S1. Measurement of physical capacity and functional tests of muscle strength, and pressure pain threshold (PPT) as a measure of pain sensitivity before (baseline) and after (post) 15 weeks of resistance exercise in fibromyalgia (FM) patients and healthy controls (CON). Data are presented as mean ±1 standard deviation (SD).

**Variables**	**CON Baseline *n* = 28**	**FM Baseline *n* = 26**	**FM vs CON Baseline P-value**	**CON Post Exercise *n* = 26**	**FM Post Exercise *n* = 25**	**FM vs CON Post Exercise *P*-Value**	**FM Baseline vs Post Exercise *P*-value**
	**Physical capacity**						
**6-minute walking test (meter)**	626.3 (±59.6)	566 (±73.4)	0.001	650 (±52.7)	571.4 (±65.3)	<0.001	0.192
**Knee extension right (Steve Strong), (kg))**	378.5 (±86.9)	323.8 (±85.5)	0.021	394.5 (±70.2)	351.4 (±105)	0.89	0.101
**Knee extension left (Steve Strong), (kg))**	351.5 (±80.3)	314.2 (±96)	0.134	360.3 (±66.0)	351.5 (±90.7)	0.693	0.029
**Handgrip fore Max Right (Grippit, (N))**	262.5 (±35.5)	175.2 (±69.9)	<0.001	277.3 (±42.7)	214.4 (±61.7)	<0.001	0.005
**Handgrip fore Max left (Grippit, (N))**	239.5 (±43.9)	180.3 (±65.9)	<0.001	256.2 (±45.0)	206.5 (±61.4)	0.002	0.037
**Elbow flexion right (Isobex, (kg))**	17.2 (±4.4)	12.2 (±4.2)	<0.001	17.6 (±4.1)	14.9 (±4.8)	0.035	0.001
**Elbow flexion Left (Isobex (kg))**	16.7 (±4.0)	12.4 (±4.5)	<0.001	17.1 (±2.8)	14.5 (±4.9)	0.024	0.012
**Location measured PPT**	**Pressure pain thresholds (PPT)**						
**Supraspinatus right**	327.8 (±119.3)	190.4 (±97.9)	<0.001	299.7 (±102.4)	214.5 (±86.1)	<0.001	0.166
**Supraspinatus left**	310.0 (±112)	201.2 (±95.6)	<0.001	314.1 (±86.5)	196.2 (±110.7)	<0.001	0.764
**Vastus lateralis right**	246.3 (±105.2)	136.3 (±72.4)	<0.001	267.3 (±112.4)	156.9 (±84.5)	<0.001	0.270
**Vastus lateralis left**	272.1 (±104.4)	147.5 (±100.3)	<0.001	255.4 (±106.4)	151.3 (±86.9)	<0.001	0.778
**Gluteus right**	369.1 (±133.9)	199.3 (±125.1)	<0.001	601.0 (±1131)	211.2 (±132)	<0.001	0.778
**Gluteus left**	397.0 (±113.5)	203.9 (±106.9)	<0.001	404.9 (±182.3)	245.6 (±166.7)	<0.001	0.271
**Knee right**	363.6 (±163.3)	168.8 (±84.7)	<0.001	346.8 (±167.0)	180.5 (±116.3)	<0.001	0.677
**Knee left**	349.4 (±158.5)	167.0 (±88.4)	<0.001	340.7 (±149.7)	184.0 (±116.3)	<0.001	0.382
**Mean PPT right side**	326.7 (±106.8)	173.7 (±80.2)	<0.001	378.7 (±318.9)	190.8 (±92.7)	0.007	0.412
**Mean PPT left side**	332.1 (±95.7)	178.6 (±95.7)	<0.001	328.7 (±111.5)	194.3 (±96.9)	<0.001	0.581

Table S2. Identified biological processes from STRING analysis tool (version 11.0). Bold marked biological processes are visualized in the protein-protein network in Figure 3.

#Term ID	Term Description	Observed Gene Count	Back-ground Gene Count	FDR	Matching Proteins in Your Network (Labels)
GO:0003012	muscle system process	9	291	6.09×10^{-8}	ACTA1,GSN,MYH7,MYOM1,MYOT,NEB, SMPX,TPM2,TTN
GO:0002576	platelet degranulation	7	129	1.45×10^{-7}	APOA1,FGA,FGB,KNG1,ORM2,PLG,TTN
**GO:0006936**	**muscle contraction**	**8**	**244**	**1.91** $\times 10^{-7}$	**ACTA1,MYH7,MYOM1,MYOT, NEB,SMPX,TPM2,TTN**
GO:0030049	muscle filament sliding	5	38	5.72×10^{-7}	ACTA1,MYH7,NEB,TPM2,TTN
GO:0003008	system process	14	1827	7.31×10^{-7}	ACTA1,FGA,FGB,GSN,KNG1,MYH7,MYOM1, MYOT,NEB,RBP4,SMPX,SYNPO,TPM2,TTN
GO:0072376	protein activation cascade	5	74	7.43×10^{-5}	C3,C4A,FGA,FGB,KNG1
GO:0045055	regulated exocytosis	9	691	1.19×10^{-5}	APOA1,C3,FGA,FGB,GSN,KNG1,ORM2, PLG,TTN
GO:0030029	actin filament-based process	8	493	1.23×10^{-5}	ACTA1,GSN,MYH7,MYOM1,MYOZ1,NEB, TPM2,TTN
GO:0050714	positive regulation of protein se-cretion	6	240	4.38×10^{-5}	FGA,FGB,MYOM1,ORM2,RBP4,TTN
GO:0030195	negative regulation of blood coag-ulation	4	48	4.46×10^{-5}	FGA,FGB,KNG1,PLG
GO:0050708	regulation of protein secretion	7	422	4.91×10^{-5}	APOA1,FGA,FGB,MYOM1,ORM2,RBP4,TTN
GO:0030239	myofibril assembly	4	59	6.81×10^{-5}	ACTA1,MYOM1,MYOZ1,TTN
GO:0032879	regulation of localization	13	2524	0.00010	APOA1,APOA4,C3,C4A,FGA,FGB,GSN,MYOM1, ORM2,PLG,RBP4,SYNPO2,TTN
GO:0048518	positive regulation of biological process	18	5459	0.00013	ACTA1,APOA1,APOA4,C3,C4A,FGA,FGB, GSN,KNG1,MYOM1,NACA,ORM2,PLG,RBP4, SYNPO,SYNPO2,TTN,UBA52
GO:0009611	response to wounding	7	547	0.00015	APOA1,FGA,FGB,GSN,KNG1,NACA,PLG
GO:0034114	regulation of heterotypic cell-cell adhesion	3	21	0.00015	APOA1,FGA,FGB

GO:0042730	fibrinolysis	3	21	0.00015	FGA,FGB,PLG
GO:0048522	positive regulation of cellular process	17	4898	0.00015	APOA1,APOA4,C3,C4A,FGA,FGB,GSN, KNG1,MYOM1,NACA,ORM2,PLG,RBP4, SYNPO,SYNPO2,TTN,UBA52
GO:0007015	actin filament organization	5	200	0.00018	ACTA1,GSN,MYOM1,TPM2,TTN
GO:0032501	multicellular organismal process	19	6507	0.00020	ACTA1,APOA1,APOA4,C3,FGA,FGB, GSN,KNG1,MYH7,MYOM1,MYOT, NACA,NEB,PLG,RBP4,SMPX,SYNPO,TPM2,TTN
GO:0014888	striated muscle adaptation	3	26	0.00021	ACTA1,GSN,MYH7
GO:0022607	cellular component assembly	12	2343	0.00021	ACTA1,APOA1,APOA4,FGA,FGB,GSN, MYOM1,MYOZ1,SYNPO,SYNPO2,TTN,UBA52
GO:0072378	blood coagulation, fibrin clot formation	3	27	0.00021	FGA,FGB,KNG1
GO:0097435	supramolecular fiber organization	6	383	0.00021	ACTA1,GSN,MYOM1,MYOZ1,TPM2,TTN
GO:0051050	positive regulation of transport	8	892	0.00022	C3,C4A,FGA,FGB,MYOM1,ORM2,RBP4,TTN
GO:0032880	regulation of protein localization	8	901	0.00023	APOA1,FGA,FGB,GSN,MYOM1, ORM2,RBP4,TTN
GO:0030036	actin cytoskeleton organization	6	418	0.00027	ACTA1,GSN,MYOM1,MYOZ1,TPM2,TTN
GO:1900026	positive regulation of substrate adhesion-dependent cell spreading	3	32	0.00027	APOA1,FGA,FGB
GO:0006941	striated muscle contraction	4	111	0.00029	MYH7,MYOM1,SMPX,TTN
GO:0070925	organelle assembly	7	666	0.00029	ACTA1,GSN,MYOM1,MYOZ1,SYNPO,SYNPO2,TTN
GO:0006959	humoral immune response	5	252	0.00030	C3,C4A,FGA,FGB,KNG1
GO:0032101	regulation of response to external stimulus	8	955	0.00030	APOA1,C3,C4A,FGA,FGB,KNG1,PLG,UBA52
GO:0090207	regulation of triglyceride metabolic process	3	34	0.00030	APOA1,APOA4,C3
GO:0016192	vesicle-mediated transport	10	1699	0.00035	APOA1,C3,FGA,FGB,GSN,KNG1,ORM2, PLG,TTN,UBA52
GO:0061061	muscle structure development	6	457	0.00035	ACTA1,MYH7,MYOM1,MYOZ1,NEB,TTN
GO:0042060	wound healing	6	461	0.00036	FGA,FGB,GSN,KNG1,NACA,PLG

GO:0014706	striated muscle tissue development	5	271	0.00038	ACTA1,MYH7,MYOM1,RBP4,TTN
GO:0051049	regulation of transport	10	1732	0.00038	APOA1,APOA4,C3,C4A,FGA,FGB,MYOM1, ORM2,RBP4,TTN
GO:0002682	regulation of immune system process	9	1391	0.00046	APOA1,C3,C4A,FGA,FGB,GSN,ORM2, RBP4,UBA52
GO:0007517	muscle organ development	5	287	0.00046	ACTA1,MYH7,MYOM1,NEB,TTN
GO:0010035	response to inorganic substance	6	491	0.00046	ACTA1,APOA4,FGA,FGB,GSN,TTN
GO:0032956	regulation of actin cytoskeleton organization	5	297	0.00053	APOA1,GSN,NEB,SYNPO,SYNPO2
**GO:0006950**	**response to stress**	**13**	3267	0.00057	APOA1,APOA4,C3,C4A,FGA,FGB,GSN, KNG1,MYH7,NACA,ORM2,PLG,UBA52
GO:0032102	negative regulation of response to external stimulus	5	310	0.00061	APOA1,FGA,FGB,KNG1,PLG
GO:0031099	regeneration	4	151	0.00062	APOA1,APOA4,GSN,NACA
GO:0030240	skeletal muscle thin filament assembly	2	6	0.00069	ACTA1,TTN
GO:0043152	induction of bacterial agglutination	2	6	0.00069	FGA,FGB
GO:0051130	positive regulation of cellular component organization	8	1128	0.00069	APOA1,C3,C4A,FGA,FGB,GSN, SYNPO,SYNPO2
GO:0016043	cellular component organization	16	5163	0.00071	ACTA1,APOA1,APOA4,C3,FGA,FGB,GSN, MYOM1,MYOZ1,NEB,PLG,SYNPO,SYNPO2, TPM2,TTN,UBA52
GO:2000257	regulation of protein activation cascade	3	54	0.00075	C3,C4A,FGA
GO:0010038	response to metal ion	5	339	0.00084	ACTA1,FGA,FGB,GSN,TTN
GO:0030300	regulation of intestinal cholesterol absorption	2	7	0.00084	APOA1,APOA4
GO:0043062	extracellular structure organization	5	339	0.00084	APOA1,APOA4,FGA,FGB,PLG
GO:2000427	positive regulation of apoptotic cell clearance	2	7	0.00084	C3,C4A

GO:0032233	positive regulation of actin filament bundle assembly	3	60	0.00093	APOA1,SYNPO,SYNPO2
GO:0010898	positive regulation of triglyceride catabolic process	2	8	0.00097	APOA1,APOA4
GO:0034371	chylomicron remodeling	2	8	0.00097	APOA1,APOA4
GO:1902905	positive regulation of supramolecular fiber organization	4	180	0.00099	APOA1,GSN,SYNPO,SYNPO2
GO:0006952	defense response	8	1234	0.0010	APOA4,C3,C4A,FGA,FGB,GSN,KNG1,ORM2
GO:0043687	post-translational protein modification	5	365	0.0010	APOA1,C3,C4A,FGA,KNG1
GO:0008015	blood circulation	5	373	0.0011	FGA,FGB,KNG1,MYH7,TTN
GO:0010810	regulation of cell-substrate adhesion	4	189	0.0011	APOA1,FGA,FGB,PLG
GO:0010873	positive regulation of cholesterol esterification	2	9	0.0011	APOA1,APOA4
GO:0030155	regulation of cell adhesion	6	623	0.0011	APOA1,FGA,FGB,GSN,KNG1,PLG
GO:0051006	positive regulation of lipoprotein lipase activity	2	9	0.0011	APOA1,APOA4
GO:0065008	regulation of biological quality	13	3559	0.0011	APOA1,APOA4,C3,FGA,FGB,GSN,KNG1, MYH7,NEB,PLG,RBP4,SYNPO,UBA52
GO:0034378	chylomicron assembly	2	10	0.0012	APOA1,APOA4
GO:0002253	activation of immune response	5	393	0.0013	C3,C4A,FGA,FGB,UBA52
GO:0051495	positive regulation of cytoskeleton organization	4	203	0.0013	APOA1,GSN,SYNPO,SYNPO2
GO:0080134	regulation of response to stress	8	1299	0.0013	APOA1,C3,C4A,FGA,FGB,KNG1, PLG,UBA52
GO:0009605	response to external stimulus	10	2152	0.0014	ACTA1,APOA1,APOA4,C3,C4A, FGA,FGB,GSN,KNG1,TTN
GO:0010737	protein kinase A signaling	2	11	0.0014	MYOM1,TTN
GO:0031639	plasminogen activation	2	11	0.0014	FGA,FGB
GO:0033700	phospholipid efflux	2	12	0.0015	APOA1,APOA4

GO:1904035	regulation of epithelial cell apoptotic process	3	78	0.0015	FGA,FGB,GSN
GO:0031347	regulation of defense response	6	676	0.0016	APOA1,C3,C4A,FGA,FGB,UBA52
GO:0045087	innate immune response	6	676	0.0016	APOA4,C3,C4A,FGA,FGB,GSN
GO:0034116	positive regulation of heterotypic cell-cell adhesion	2	13	0.0017	FGA,FGB
GO:0034380	high-density lipoprotein particle assembly	2	13	0.0017	APOA1,APOA4
GO:0051258	protein polymerization	3	83	0.0017	FGA,FGB,GSN
GO:0071688	striated muscle myosin thick filament assembly	2	13	0.0017	MYOM1,TTN
GO:0050790	regulation of catalytic activity	10	2249	0.0018	APOA1,APOA4,C3,C4A,GSN,KNG1, SYNPO2,TPM2,TTN,UBA52
GO:0001523	retinoid metabolic process	3	87	0.0019	APOA1,APOA4,RBP4
GO:0002224	toll-like receptor signaling pathway	3	87	0.0019	FGA,FGB,UBA52
GO:0110053	regulation of actin filament organization	4	235	0.0020	APOA1,GSN,SYNPO,SYNPO2
GO:0051239	regulation of multicellular organismal process	11	2788	0.0021	APOA1,APOA4,C3,FGA,FGB,KNG1, MYH7,NACA,ORM2,PLG,RBP4
GO:0045723	positive regulation of fatty acid biosynthetic process	2	16	0.0022	APOA1,APOA4
GO:0051128	regulation of cellular component organization	10	2306	0.0022	APOA1,APOA4,C3,C4A,FGA,FGB, GSN,NEB,SYNPO,SYNPO2
GO:0090066	regulation of anatomical structure size	5	464	0.0022	FGA,FGB,GSN,KNG1,NEB
GO:0090277	positive regulation of peptide hormone secretion	3	95	0.0022	FGA,FGB,RBP4
GO:0034375	high-density lipoprotein particle remodeling	2	17	0.0024	APOA1,APOA4
GO:0043691	reverse cholesterol transport	2	17	0.0024	APOA1,APOA4
GO:0051241	negative regulation of multicellular organismal process	7	1098	0.0025	APOA1,APOA4,FGA,FGB,KNG1,PLG,RBP4
GO:0032103	positive regulation of response to	5	499	0.0029	C3,FGA,FGB,PLG,UBA52

	external stimulus				
GO:0065003	protein-containing complex assembly	8	1514	0.0029	APOA1,APOA4,FGA,FGB,GSN, MYOM1,TTN,UBA52
GO:0002250	adaptive immune response	4	280	0.0033	C3,C4A,FGA,FGB
GO:0044057	regulation of system process	5	516	0.0033	APOA1,APOA4,FGA,FGB,MYH7
GO:0006810	transport	13	4130	0.0034	APOA1,APOA4,C3,FGA,FGB,GSN, KNG1,NACA,ORM2,PLG,RBP4,TTN,UBA52
**GO:0006955**	**immune response**	**8**	**1560**	**0.0034**	**APOA4,C3,C4A,FGA,FGB,GSN,KNG1,ORM2**
GO:0007596	blood coagulation	4	288	0.0035	FGA,FGB,KNG1,PLG
GO:0032268	regulation of cellular protein metabolic process	10	2486	0.0035	APOA1,C3,C4A,FGA,FGB,GSN,KNG1, SYNPO2,TTN,UBA52
GO:0051707	response to other organism	7	1173	0.0035	APOA4,C3,C4A,FGA,FGB,GSN,KNG1
GO:0070613	regulation of protein processing	3	116	0.0035	C3,C4A,GSN
GO:0034622	cellular protein-containing complex assembly	6	832	0.0036	FGA,FGB,GSN,MYOM1,TTN,UBA52
GO:0033344	cholesterol efflux	2	24	0.0038	APOA1,APOA4
GO:0010954	positive regulation of protein processing	2	25	0.0040	C3,GSN
GO:0070527	platelet aggregation	2	25	0.0040	FGA,FGB
GO:0031102	neuron projection regeneration	2	26	0.0042	APOA1,APOA4
GO:0035296	regulation of tube diameter	3	129	0.0042	FGA,FGB,KNG1
GO:0050776	regulation of immune response	6	873	0.0042	APOA1,C3,C4A,FGA,FGB,UBA52
**GO:0050896**	**response to stimulus**	**18**	**7824**	**0.0042**	**ACTA1,APOA1,APOA4,C3,C4A,FGA, FGB,GSN,KNG1,MYH7,MYOM1,NACA, ORM2,PLG,RBP4,SYNPO,TTN,UBA52**
GO:0051336	regulation of hydrolase activity	7	1238	0.0042	APOA1,APOA4,C3,C4A,GSN,KNG1,TPM2
GO:0051592	response to calcium ion	3	129	0.0042	FGA,FGB,TTN
GO:1905952	regulation of lipid localization	3	129	0.0042	APOA1,APOA4,C3
GO:0002684	positive regulation of immune system process	6	882	0.0043	C3,C4A,FGA,FGB,RBP4,UBA52
GO:0003300	cardiac muscle hypertrophy	2	28	0.0044	MYH7,TTN
GO:0015850	organic hydroxy compound transport	3	134	0.0044	APOA1,APOA4,RBP4

GO:2000352	negative regulation of endothelial cell apoptotic process	2	28	0.0044	FGA,FGB
GO:0097746	regulation of blood vessel diameter	3	137	0.0046	FGA,FGB,KNG1
GO:0006911	phagocytosis, engulfment	2	31	0.0051	C3,GSN
GO:0019730	antimicrobial humoral response	3	143	0.0051	FGA,FGB,KNG1
GO:0045907	positive regulation of vasoconstriction	2	33	0.0057	FGA,FGB
GO:0070328	triglyceride homeostasis	2	33	0.0057	APOA1,APOA4
GO:0009893	positive regulation of metabolic process	11	3280	0.0058	ACTA1,APOA1,APOA4,C3,FGA, FGB,GSN,MYOM1,NACA,TTN,UBA52
GO:0006958	complement activation, classical pathway	2	34	0.0059	C3,C4A
GO:0048738	cardiac muscle tissue development	3	154	0.0059	MYH7,RBP4,TTN
GO:0002697	regulation of immune effector process	4	362	0.0061	APOA1,C3,C4A,RBP4
GO:0061384	heart trabecula morphogenesis	2	35	0.0061	NACA,RBP4
GO:0001932	regulation of protein phosphorylation	7	1370	0.0063	APOA1,C3,FGA,FGB,SYNPO2,TTN,UBA52
GO:0022407	regulation of cell-cell adhesion	4	366	0.0063	APOA1,FGA,FGB,PLG
GO:0031349	positive regulation of defense response	4	365	0.0063	C3,FGA,FGB,UBA52
GO:1902042	negative regulation of extrinsic apoptotic signaling pathway via death domain receptors	2	36	0.0063	FGA,FGB
GO:0050706	regulation of interleukin-1 beta secretion	2	37	0.0066	APOA1,ORM2
GO:0045214	sarcomere organization	2	38	0.0068	MYOM1,TTN
GO:0051180	vitamin transport	2	38	0.0068	APOA1,RBP4
GO:0003205	cardiac chamber development	3	166	0.0069	MYH7,NACA,RBP4
GO:0002758	innate immune response-activating signal transduction	3	168	0.0070	FGA,FGB,UBA52

GO:0006695	cholesterol biosynthetic process	2	41	0.0076	APOA1,APOA4
GO:0046890	regulation of lipid biosynthetic process	3	174	0.0076	APOA1,APOA4,C3
GO:0052548	regulation of endopeptidase activity	4	393	0.0076	C3,C4A,GSN,KNG1
GO:0002376	immune system process	9	2370	0.0078	APOA4,C3,C4A,FGA,FGB,GSN, KNG1,ORM2,UBA52
GO:0048747	muscle fiber development	2	43	0.0080	ACTA1,TTN
GO:0048468	cell development	7	1493	0.0092	ACTA1,APOA1,APOA4,GSN, MYOM1,MYOZ1,TTN
GO:0019538	protein metabolic process	12	4194	0.0102	APOA1,APOA4,C3,C4A,FGA, FGB,GSN,KNG1,NACA,PLG,TTN,UBA52
GO:0044267	cellular protein metabolic process	11	3603	0.0104	APOA1,APOA4,C3,C4A,FGA, GSN,KNG1,NACA,PLG,TTN,UBA52
GO:0051346	negative regulation of hydrolase activity	4	438	0.0104	APOA1,C3,C4A,KNG1
GO:0030449	regulation of complement activation	2	52	0.0107	C3,C4A
GO:0051704	multi-organism process	9	2514	0.0109	APOA4,C3,C4A,FGA,FGB, GSN,KNG1,PLG,UBA52
GO:0042246	tissue regeneration	2	54	0.0113	GSN,NACA
GO:0048584	positive regulation of response to stimulus	8	2054	0.0123	APOA1,C3,C4A,FGA,FGB,GSN,PLG,UBA52
GO:0060627	regulation of vesicle-mediated transport	4	480	0.0138	C3,C4A,FGA,FGB
GO:0006954	inflammatory response	4	482	0.0140	C3,C4A,KNG1,ORM2
GO:0009888	tissue development	7	1626	0.0140	ACTA1,GSN,MYH7,MYOM1,NACA,RBP4,TTN
GO:0007507	heart development	4	485	0.0141	MYH7,NACA,RBP4,TTN
GO:0010647	positive regulation of cell communication	7	1631	0.0141	APOA1,C3,FGA,FGB,GSN,RBP4,UBA52
GO:0006508	proteolysis	6	1203	0.0142	C3,C4A,FGA,FGB,PLG,UBA52
GO:0023056	positive regulation of signaling	7	1638	0.0142	APOA1,C3,FGA,FGB,GSN,RBP4,UBA52

GO:0055008	cardiac muscle tissue morphogenesis	2	63	0.0144	MYH7,TTN
GO:0003007	heart morphogenesis	3	235	0.0149	MYH7,RBP4,TTN
GO:0044089	positive regulation of cellular component biogenesis	4	498	0.0150	APOA1,GSN,SYNPO,SYNPO2
GO:0048646	anatomical structure formation involved in morphogenesis	5	831	0.0150	ACTA1,MYOM1,MYOZ1,RBP4,TTN
GO:0010951	negative regulation of endopeptidase activity	3	242	0.0158	C3,C4A,KNG1
GO:0048523	negative regulation of cellular process	12	4454	0.0158	APOA1,APOA4,C3,C4A,FGA,FGB, GSN,KNG1,NACA,PLG,RBP4,UBA52
GO:0007162	negative regulation of cell adhesion	3	245	0.0162	APOA1,KNG1,PLG
GO:0010817	regulation of hormone levels	4	511	0.0162	APOA1,FGA,FGB,RBP4
GO:0065007	biological regulation	21	11740	0.0168	ACTA1,APOA1,APOA4,C3,C4A,FGA, FGB,GSN,KNG1,MYH7,MYOM1,NACA,NEB, ORM2,PLG,RBP4,SYNPO, SYNPO2,TPM2, TTN,UBA52
GO:1903522	regulation of blood circulation	3	249	0.0168	FGA,FGB,MYH7
GO:0042632	cholesterol homeostasis	2	71	0.0169	APOA1,APOA4
GO:0046470	phosphatidylcholine metabolic process	2	72	0.0172	APOA1,APOA4
GO:0060048	cardiac muscle contraction	2	73	0.0176	MYH7,TTN
GO:0045921	positive regulation of exocytosis	2	78	0.0192	FGA,FGB
GO:0051492	regulation of stress fiber assembly	2	78	0.0192	APOA1,SYNPO
GO:1901564	organonitrogen compound metabolic process	13	5281	0.0195	APOA1,APOA4,C3,C4A,FGA,FGB, GSN,KNG1,MYH7,NACA,PLG,TTN,UBA52
GO:0006869	lipid transport	3	272	0.0197	APOA1,APOA4,RBP4
GO:0006928	movement of cell or subcellular component	6	1355	0.0221	ACTA1,APOA1,MYH7,NEB,TPM2,TTN
GO:0010628	positive regulation of gene expression	7	1826	0.0225	ACTA1,C3,GSN,MYOM1,NACA,TTN,UBA52
GO:0006066	alcohol metabolic process	3	290	0.0229	APOA1,APOA4,RBP4

GO:0022603	regulation of anatomical structure morphogenesis	5	961	0.0240	APOA1,C3,FGA,FGB,NACA
GO:0030198	extracellular matrix organization	3	296	0.0240	FGA,FGB,PLG
GO:0090130	tissue migration	2	90	0.0240	ACTA1,APOA1
GO:0002673	regulation of acute inflammatory response	2	92	0.0248	C3,C4A
GO:0050789	regulation of biological process	20	11116	0.0249	ACTA1,APOA1,APOA4,C3,C4A,FGA,FGB, GSN,KNG1,MYH7,MYOM1,NACA,NEB, ORM2,PLG,RBP4,SYNPO,SYNPO2,TTN,UBA52
GO:0002443	leukocyte mediated immunity	4	632	0.0291	C3,C4A,GSN,ORM2
GO:0001775	cell activation	5	1024	0.0303	C3,FGA,FGB,GSN,ORM2
GO:0043900	regulation of multi-organism process	4	653	0.0321	FGA,FGB,GSN,UBA52
GO:0048585	negative regulation of response to stimulus	6	1483	0.0321	APOA1,FGA,FGB,KNG1,PLG,UBA52
GO:0009967	positive regulation of signal transduction	6	1493	0.0329	APOA1,C3,FGA,FGB,GSN,UBA52
GO:0050727	regulation of inflammatory response	3	338	0.0329	APOA1,C3,C4A
GO:0010604	positive regulation of macromolecule metabolic process	9	3081	0.0330	ACTA1,C3,FGA,FGB,GSN,MYOM1,NACA, TTN,UBA52
GO:0009653	anatomical structure morphogenesis	7	1992	0.0333	ACTA1,MYH7,MYOM1,MYOZ1,NACA,RBP4,TTN
GO:0042981	regulation of apoptotic process	6	1501	0.0333	FGA,FGB,GSN,KNG1,NACA,UBA52
GO:0042157	lipoprotein metabolic process	2	114	0.0349	APOA1,APOA4
GO:0006996	organelle organization	9	3131	0.0359	ACTA1,GSN,MYOM1,MYOZ1,SYNPO, SYNPO2,TPM2,TTN,UBA52
GO:0007160	cell-matrix adhesion	2	119	0.0376	FGA,FGB
GO:0051240	positive regulation of multicellular organismal process	6	1551	0.0377	C3,FGA,FGB,NACA,ORM2,PLG
GO:0007519	skeletal muscle tissue development	2	122	0.0388	ACTA1,MYOM1
GO:0002700	regulation of production of molecular	2	124	0.0397	APOA1,RBP4

	mediator of immune response				
GO:0019222	regulation of metabolic process	14	6516	0.0397	ACTA1,APOA1,APOA4,C3,C4A,FGA, FGB,GSN,KNG1,MYOM1,NACA,SYNPO2, TTN,UBA52
GO:0071347	cellular response to interleukin-1	2	124	0.0397	FGB,UBA52
GO:0003231	cardiac ventricle development	2	125	0.0400	MYH7,NACA
GO:0016202	regulation of striated muscle tissue development	2	127	0.0408	NACA,RBP4
GO:0003206	cardiac chamber morphogenesis	2	128	0.0410	MYH7,RBP4
GO:0045807	positive regulation of endocytosis	2	129	0.0414	C3,C4A
GO:2001233	regulation of apoptotic signaling pathway	3	388	0.0429	FGA,FGB,GSN
GO:0045471	response to ethanol	2	134	0.0437	GSN,RBP4
GO:0048583	regulation of response to stimulus	10	3882	0.0437	APOA1,C3,C4A,FGA,FGB,GSN, KNG1,MYH7,PLG,UBA52
GO:0043902	positive regulation of multi-organism process	3	394	0.0440	FGA,FGB,UBA52
